# Authors’ Reply: Understanding the Impact of Social Media Information and Misinformation Producers on Health Information Seeking. Comment on “Health Information Seeking Behaviors on Social Media During the COVID-19 Pandemic Among American Social Networking Site Users: Survey Study”

**DOI:** 10.2196/31569

**Published:** 2022-02-04

**Authors:** Stephen Neely, Christina Eldredge, Ronald Sanders

**Affiliations:** 1 School of Public Affairs University of South Florida Tampa, FL United States; 2 School of Information University of South Florida Tampa, FL United States; 3 Florida Center for Cybersecurity School of Information University of South Florida Tampa, FL United States

**Keywords:** social media, internet, communication, public health, COVID-19, usage, United States, information seeking, web-based health information, online health information, survey, mistrust, vaccination, misinformation

We appreciate Boudreau and colleagues’ [1] thoughtful consideration of our recent survey study [2], which examined American people’s use of social networking sites (SNS) to learn and stay informed about the COVID-19 pandemic. As they point out, we surveyed a representative sample of American adults (N=1003) and found that most SNS users had not fact-checked COVID-19–related information with a medical professional, and those who had opted to follow credible, scientific sources on social media were significantly more likely to undergo vaccination [2]. In reply, Boudreau and colleagues noted that our study—and others like it—has focused primarily on consumers rather than the producers and publishers of medical content on social media [1]. They propose that researchers should shift their focus “from the consumers to the producers of this information,” and, in particular, they emphasize the possibility of developing tools to assess and classify health-related posts on social media in order to help consumers distinguish medically valid guidance from potential misinformation.

We understand and affirm the underlying spirit of Boudreau et al’s [1] recommendation, and building on that, we would endorse an “all of the above” approach to the study of social media moving forward. A comprehensive research agenda—drawing on a diverse range of perspectives and methodological techniques—will be needed in order to understand and keep pace with social media’s growing and evolving role in health information seeking. This includes greater attention to issues of content and publisher credibility, as the authors suggest, though it should be noted that social media often obscures the distinction between publishers and consumers [3]. It also means that health professionals will need to gain awareness of and interpret emerging techniques in data mining, natural language processing, and network analysis. These are essential to identifying influential network nodes and understanding how health information spreads in complex social networks. For reference, we conducted a similar analysis during the 2015-2016 Zika virus outbreak [4].

However, in pursuing a comprehensive research agenda around social media, it is critical that researchers not lose sight of the consumer perspective. We agree that promoting and affirming accuracy “at the source” is critical, but so too is understanding which sources of health information consumers encounter, trust, and rely on. Unfortunately, recent studies have noted declining trust in science among many Americans, including the Institution of Medicine [5,6]. This is especially salient in the case of politicized public health emergencies, such as the COVID-19 pandemic. Add to this the politicization and fragmentation of social media platforms themselves, and we find ourselves immersed in an information environment where even quality markers are often interpreted as political statements. While health professionals are not to blame for these trends, it is nonetheless important that they be aware of and responsive to them. This means that it is critical for research and scholars to stay focused on understanding consumer-level preferences, behaviors, and outcomes while also working to improve health messaging at its source.

## Abbreviations

SNS: social networking sites
